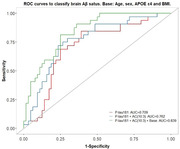# Plasma metabolites associated with plasma P‐tau181 in preclinical Alzheimer's disease

**DOI:** 10.1002/alz.091167

**Published:** 2025-01-09

**Authors:** Tahmida Sharmin, James D. Doecke, Pratishtha Chatterjee, Kevin Huynh, Benjamin Heng, Shaun Eslick, Manohar Garg, Ralph N Martins

**Affiliations:** ^1^ Macquarie University, Sydney, NSW Australia; ^2^ The Australian e‐Health Research Centre, CSIRO, Brisbane, QLD Australia; ^3^ Baker Heart and Diabetes Institute, Melbourne, VIC Australia; ^4^ Edith Cowan University, Perth, Western Australia Australia

## Abstract

**Background:**

Alzheimer's disease (AD) pathogenesis is not restricted to amyloid‐beta, Aβ, and tau pathologies but involves dysregulation in diverse cellular and molecular processes. Numerous metabolomic studies revealed plasma metabolite alterations in AD individuals compared to healthy controls. Nevertheless, plasma P‐tau181, an established biomarker for AD diagnosis and prognosis, has been described to reflect initial multiple cortical region Aβ deposition in cognitively intact adults. The current study aims to identify plasma metabolites associated with plasma P‐tau181 at the preclinical stage and better understand the associated biochemical mechanisms for AD pathogenesis.

**Method:**

In the current study, 100 older adults with no objective cognitive impairment, MoCA and MMSE ≥ 26, from the Kerr Anglican Retirement Village Initiative in Ageing Health (KARVIAH) cohort, comprising 65 CI Aβ‐ (cognitively intact normal brain Aβ) and 35 CI Aβ+ (cognitively intact higher brain Aβ) individuals, were assessed for plasma P‐tau181, via ultra‐sensitive Quanterix Simoa technology, and plasma metabolites, via mass spectrometry‐based BIOCRATES kit, and then investigated for associations, both before and after adjusting for confounding variables, in the study groups. Additionally, P‐tau181‐associated plasma metabolites were evaluated using the receiver operating characteristic (ROC) curves for the potential to classify brain Aβ status.

**Result:**

In the entire cohort, significant positive associations of plasma metabolites, including acylcarnitines, amino acid citrulline, and three biogenic amines (creatinine, kynurenine and SDMA), were observed with P‐tau181 and similar associations, except for kynurenine, were detected in CI Aβ‐. In contrast, in CI Aβ+, only acylcarnitine, AC(10:3), was found to have a positive association with P‐tau181 and further, upon including AC(10:3), the AUC for P‐tau181 (AUC=70.9%) potentially outperformed (AUC=76.2%), which additionally topped to 83.9% when combined with a base model (Abstract Figure).

**Conclusion:**

These findings suggest that the higher the plasma P‐tau181, the higher the medium chain acylcarnitine, AC(10:3), in plasma in cognitively intact older adults at risk for AD, indicating a link between early Aβ pathology and fatty acid oxidation mediated energy metabolism pathway. Additionally, associated metabolite strengthens the significance of P‐tau181 in classifying brain Aβ status in cognitively intact older adults. Therefore, plasma P‐tau181‐associated plasma metabolite may serve as potential predictive marker for preclinical AD pathogenesis.